# Supplementary material for: Anxious about rejection, avoidant of neglect: Infant marmosets tune their attachment based on individual caregiver’s parenting style
Source: Commun Biol. 2024 Feb 20;7:212. doi: 10.1038/s42003-024-05875-6 (PMC10879543; doi:10.1038/s42003-024-05875-6)
Supplement: Supplementary file 2 — Supplementary Information [file 42003_2024_5875_MOESM2_ESM.pdf]

Supplementary Information

Title

Anxious about rejection, avoidant of neglect: Infant marmosets tune their attachment based on individual caregiver's parenting style

Authors

Saori Yano-Nashimoto[1,2#], Anna Truzzi[1,3,4#], Kazutaka Shinozuka[1#], Ayako Y. Murayama[1,5,6,&], Takuma Kurachi[1,7], Keiko Moriya-Ito[8], Hironobu Tokuno[8‡], Eri Miyazawa[1], Gianluca Esposito[1,4], Hideyuki Okano[5,6], Katsuki Nakamura[9], Atsuko Saito[1,10*], Kumi O. Kuroda[1,11*]

Affiliations

1 Laboratory for Affiliative Social Behavior, RIKEN Center for Brain Science, Wako, Japan.

2 Laboratory of Physiology, Department of Basic Veterinary Sciences, Graduate School of Veterinary Medicine, Hokkaido University, Sapporo, Japan.

3 Trinity College Institute of Neuroscience, School of Psychology, Trinity College Dublin, Dublin, Ireland.

4 Department of Psychology and Cognitive Science, University of Trento, Rovereto, TN, Italy.

5 Department of Physiology, Keio University School of Medicine, Shinjuku-ku, Japan.

6 Laboratory for Marmoset Neural Architecture, RIKEN Center for Brain Science, Wako, Japan.

7 Department of Agriculture, Tokyo University of Agriculture and Technology, Fuchu, Japan

8 Department of Brain & Neurosciences, Tokyo Metropolitan Institute of Medical Science, Setagaya-ku, Japan.

9 Center for the Evolutionary Origins of Human Behavior, Kyoto University, Inuyama, Japan.

10 Department of Psychology, Sophia University, Chiyoda-ku, Japan.

11 Kuroda Laboratory, School of Life Science and Technology, Tokyo Institute of Technology, Yokohama, Japan

# These authors contributed equally to this study.

‡ The author is deceased.
& Current address: Neural Circuit Unit, Okinawa Institute Science and Technology Graduate University, Onna, Japan

*Corresponding author:
Kumi O. Kuroda
Kuroda Laboratory, School of Life Science and Technology, Tokyo Institute of Technology
Nagatsuta-cho 4259, Midori-ku, Yokohama-shi, Kanagawa 226-0026, Japan
E-mail: kurodalab@bio.titech.ac.jp

Atsuko Saito
Department of Psychology, Faculty of Human Sciences, Sophia University,
Kioi-cho 7-1, Chiyoda-ku, Tokyo 102-8554, Japan
E-mail: atsaito@sophia.ac.jp

Supplementary Figures

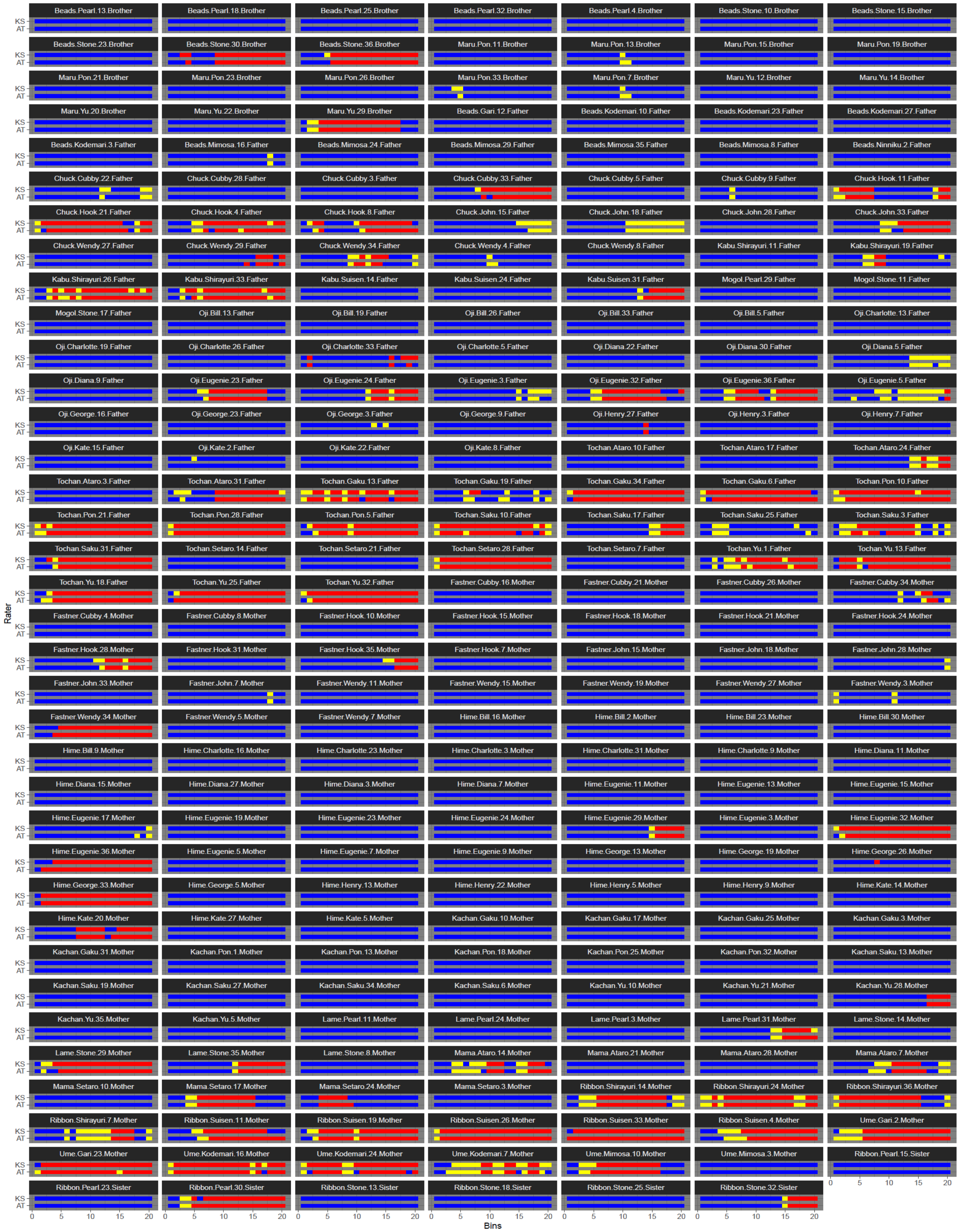

Supplementary Figure 1 Inter-observational reliability between the on-site behavioral coding and off-site, detailed behavioral microanalyses of the infant retrieval assay.

The on-site analyses performed with 30-sec bins (by rater S.K. [37]) are compared with the off-site analyses with 0.2-sec bins in this study (by rater A.T.), by converting A.T. ‘s coding results into the 30-sec bins and classifying them as “carried” (blue), “rejected” (yellow), or “not carried” (red). The interrater reliability between the two raters was assessed by Cohen’s kappa ($\kappa = 0.926$, $p < 0.001$).

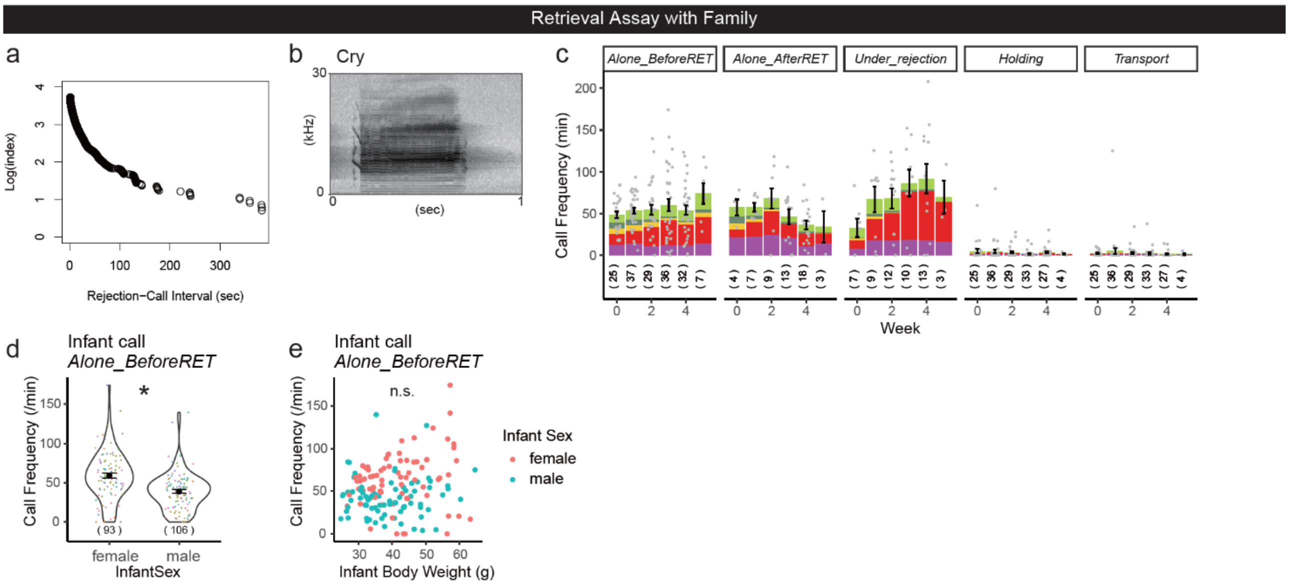

Retrieval Assay with Family
a
Log(index)
Rejection−Call Interval (sec)
b
Cry
(kHz)
(sec)
c
Alone_BeforeRET
Alone_AfterRET
Under_rejection
Holding
Transport
Call Frequency (min)
Week
d
Infant call
Alone_BeforeRET
Call Frequency (/min)
female
male
InfantSex
e
Infant call
Alone_BeforeRET
n.s.
Call Frequency (/min)
Infant Body Weight (g)
Infant Sex
female
male

Supplementary Figure 2 Infant behaviors in the retrieval assay using family-reared infants.

a Segmented regression analysis of the rejection-call interval. The inflection point could not be determined; thus, we also used the 9.4-second offset obtained from movement analysis (Fig. 2d) for call analysis. A total of 77.18% (3981 out of 5158) of the infant calls during carrying occurred during and within 9.4 sec after the preceding rejection.

b Spectrogram of the infant's cry call.

c Developmental changes in infant call frequencies in each social context. The columns and bars indicate the mean ± s.e. of total calls. The color shows the type of call. This graph corresponds with Fig. 2j. Only the data with detailed vocal analysis were included. The numbers within the parentheses are the numbers of the sessions. Each dot shows the total call frequency in each session. Data was collected from 166 sessions of 35 dyads.

d Violin plot of the call frequency of male and female infants during *Alone_BeforeRET*. The numbers within parentheses are the numbers of the trials. As the only sex difference identified in infant attachment behaviors (Fig. 1c), female infants called more than male infants when the infants were alone before the first retrieval (GLMM). This effect was not attributed to the sexual difference in body size growth, as there was no correlation between the isolation calls and body weight (Supplementary Figure 2e). (199 sessions of 55 dyads)

e Scatter plot of body weight and infants' call frequency during *Alone_BeforeRET*. The call frequency was not affected by body weight (GLMM). Female: orange, male: green. (155 sessions of 50 dyads)

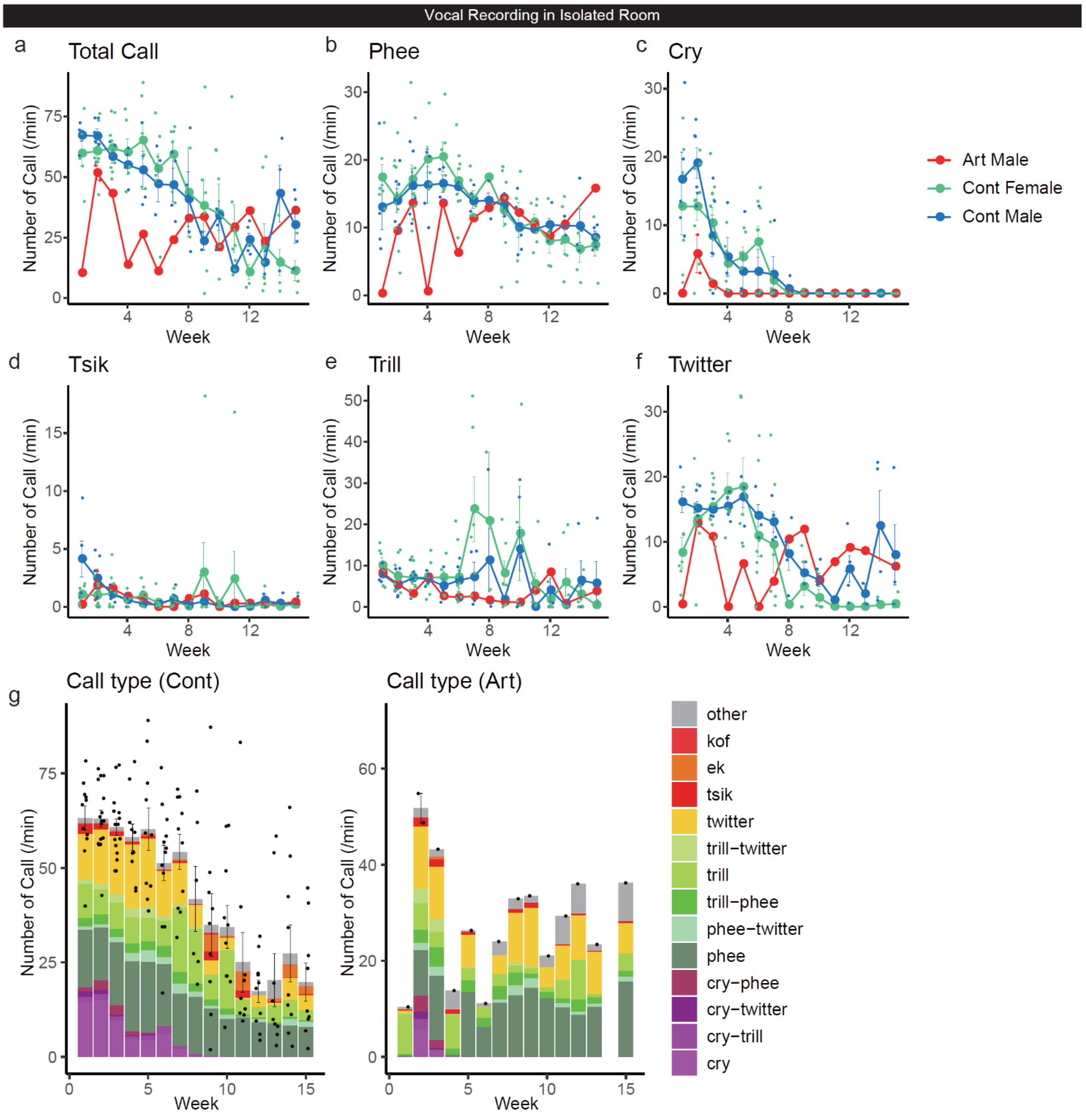

Supplementary Figure 3 Vocal behaviors in isolation.

a-f The mean ± s.e. call frequencies in the isolated recordings (Art: n = 1 male; Cont: n = 9 (4 males, 5 females). Total calls (a) as well as phee (b), cry (c), tsik (d), trill (e), and twitter calls (f). Each dot shows the value in each session.

g The mean ± s.e. total calls and composition of call types of Cont (left) and Art infants (right). Error bars represent the standard error of the total calls. Each dot shows the number of total calls in each session. The left is the same as in Fig. 2l, shown for comparison.

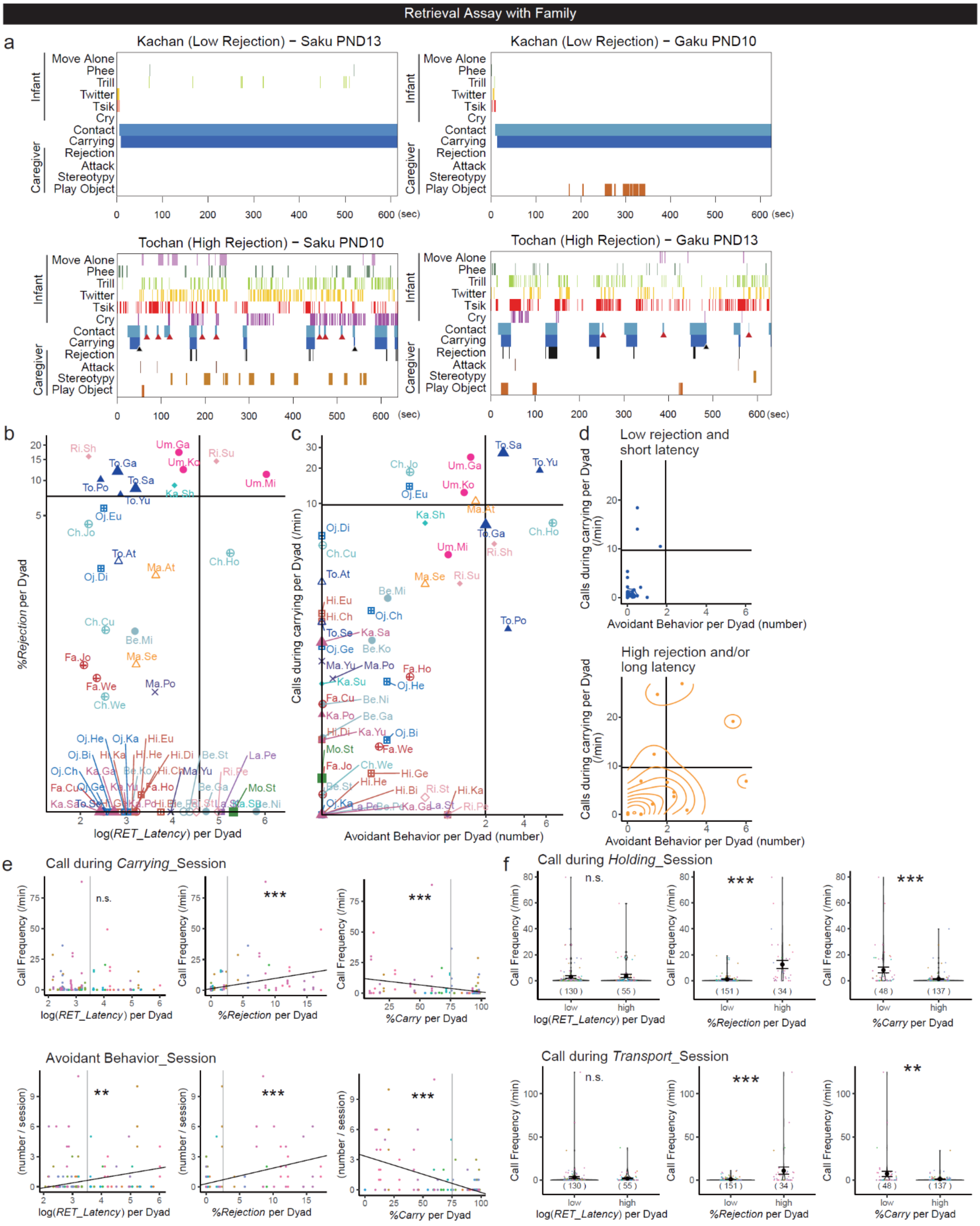

Retrieval Assay with Family
a
Kachan (Low Rejection) − Saku PND13
Kachan (Low Rejection) − Gaku PND10
Tochan (High Rejection) − Saku PND10
Tochan (High Rejection) − Gaku PND13
Infant
Caregiver
Move Alone
Phee
Trill
Twitter
Tsik
Cry
Contact
Carrying
Rejection
Attack
Stereotypy
Play Object
b
%Rejection per Dyad
log(RET_Latency) per Dyad
c
Calls during carrying per Dyad (/min)
Avoidant Behavior per Dyad (number)
d
Low rejection and short latency
High rejection and/or long latency
Calls during carrying per Dyad (/min)
Avoidant Behavior per Dyad (number)
e
Call during Carrying_Session
Call Frequency (/min)
log(RET_Latency) per Dyad
%Rejection per Dyad
%Carry per Dyad
n.s.
***
Avoidant Behavior_Session
(number / session)
**
f
Call during Holding_Session
Call during Transport_Session
low
high

Supplementary Figure 4 Relationship between caregiver and infant behaviors in the retrieval assay using family-reared infants.

a Representative raster plots of the retrieval assay using a pair of littermates and their parent. See text for explanation. Black triangles: dismounting without preceding rejection within 9.4 sec. Red triangles: refusal to cling when contacted. Left two plots were also shown in Fig. 3a-b.

b-c Variation in caregiving (b) and attachment behavior (c) during postnatal weeks 0-3 (54 dyads, excluding one dyad in which the caregiver never retrieved the infant). Each point represents the value averaged for each caregiver-infant dyad, with the color of each caregiver (warm colors: females, cold colors: males). The marker shapes indicate each breeding pair. Labels near markers are the first two letters of the caregiver's and infant's names. For example, dark blue for Tochan, pink triangles for Kachan. The vertical and horizontal lines show the mean + 1 s.d.

d Frequency of infant calls during carrying and the number of avoidant behaviors averaged for each dyad. Upper: both *%Rejection* and the log of *RET_Latency* were less than the mean + 1 s.d. (n = 36), lower: the other dyads (n = 18). Two-dimensional probability density was overlaid as a contour with 10 bins. Vertical and horizontal lines indicate the mean + 1 s.d. for each axis.

e Scatter plots and regression lines of infant calls during carrying (top) and avoidant behavior (bottom) and caregiving parameters (left: *RET_Latency*, center: *%Rejection*, right: *%Carrying*). More avoidant behaviors were observed in the dyad with longer retrieval latency (t = 2.76, p = 0.0078), although the infant calls during carrying were not different from those in the other dyad. In the dyad with a higher %Rejection, the infant calls during carrying and the avoidant behaviors were more frequent (call-during-carrying: t = 5.13, $p < 0.001$, avoidant behavior: t = 4.323, $p < 0.001$). In the dyad with a higher %Carrying than the average, the infant calls during carrying and the avoidant behaviors were less frequent (call-during-carrying: t = -4.25, $p < 0.001$, avoidant behavior: t = -7.367, $p < 0.001$). Each dot shows the value of each session, and the same color indicates data from the same caregiver. (199 sessions of 55 dyads. For %Rejection and call during carrying, 185 sessions of 54 dyads, as sessions without first retrieval were excluded.)

f Violin plots of the infant calls during holding (top) and transport (bottom) in the groups of dyads divided by high/low parental behaviors (*RET_Latency*, *%Rejection*, *%Carrying*). Each dot shows the value of each session, and the color indicates the individual caregiver. The filled circles and error bars show the mean ± s.e. The numbers within parentheses are the numbers of the sessions. (185 sessions of 54 dyads, as sessions without first retrieval were excluded.)

GLMMs were used for statistical analyses. **: $p < 0.01$, ***: $p < 0.001$

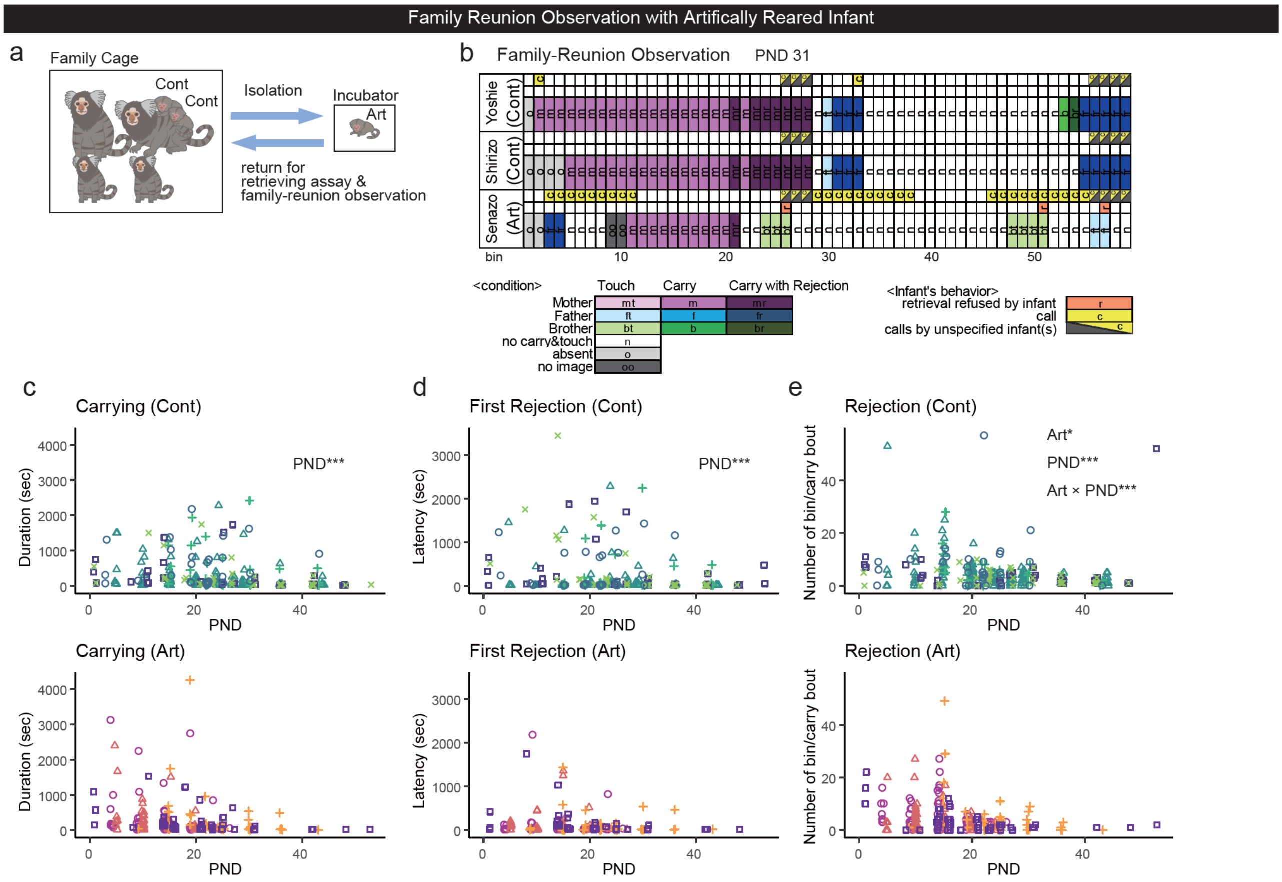

Family Reunion Observation with Artifically Reared Infant
a
Family Cage
Cont
Cont
Isolation
Incubator
Art
return for
retrieving assay &
family-reunion observation
b
Family-Reunion Observation
PND 31
Yoshie (Cont)
Shirizo (Cont)
Senazo (Art)
bin
<condition>
Touch
Carry
Carry with Rejection
Mother
Father
Brother
no carry&touch
absent
no image
<Infant's behavior>
retrieval refused by infant
call
calls by unspecified infant(s)
c
Carrying (Cont)
PND***
Duration (sec)
PND
Carrying (Art)
d
First Rejection (Cont)
PND***
Latency (sec)
First Rejection (Art)
e
Rejection (Cont)
Art*
PND***
Art × PND***
Number of bin/carry bout
Rejection (Art)

Supplementary Figure 5 Family reunion observation with artificially reared infants.

A Schematic of the behavioral tests of artificially reared (Art) and control (Cont) infants.

B Representative raster plot of family reunion observation. One Art and two Cont littermate infants at PND 31 were returned to their family cages one by one. The Art infant Senazo (the bottom row) was first retrieved by the father but soon dismounted because of the father's rejection (dark blue bins). The experimenter assisted Senazo in clinging to the mother, which was tolerant and was carrying two Cont infants (Yoshie and Shirizo, the top and middle row). The mother carried three infants (10th to 20th pink bins) and then rejected them (the 21st dark purple bin). Senazo dismounted, while two Cont littermates kept clinging even though the mother kept rejecting them for 6 consecutive bins. In the meantime, Senazo came into contact with the brother (24th-26th light green bins) but did not cling to the brother (retrieval refused by the infant, orange mark above the bin). Senazo called in two long bouts from the 29th and 46th bins (yellow marks), and came into contact with the brother and the father sequentially but refused to cling. The two Cont infants did not call while being alone after dismounted from the father at the 34th bin, which showed age-appropriate maturity. In contrast, Senazo showed excessive distress calls (which should attract caregivers) but avoided simultaneously, the behavior reminiscent of "disorganized" attachment in humans. In addition, compared to Cont littermates, Senazo tended to give up clinging and dismount easily by brief rejection. Bin: 10-sec.

c-e The duration of a carrying bout (c), the latency of the first rejection (d), and the number of caregivers' rejection bins (e) in a carrying bout were calculated. A carrying bout is a period from the bin when an infant starts to cling onto a caregiver to the bin when the infant dismounts from the caregiver or moves to another caregiver. The shape and color of the markers represent each infant.

c The duration of a carrying bout (Cont: 363 bouts, Art: 299 bouts) was shorter among the older infants (t = -5.63, $p < 0.001$).

d The latency of the first rejection in a carrying bout (Cont: 323 bouts, Art: 244 bouts) was shorter among the older infants (t = -5.10, $p < 0.001$).

e In the number of caregiver rejection bins (Cont: 363 bouts, Art: 299 bouts), the interaction effect of Art and PND was significant (z = -7.65, $p < 0.001$), indicating that the coefficient of PND among the artificially reared infants was smaller than that among the family-reared infants.

Statistical analyses were performed by the generalized linear mixed model (GLMM). *: $p < 0.05$, **: $p < 0.01$, ***: $p < 0.001$

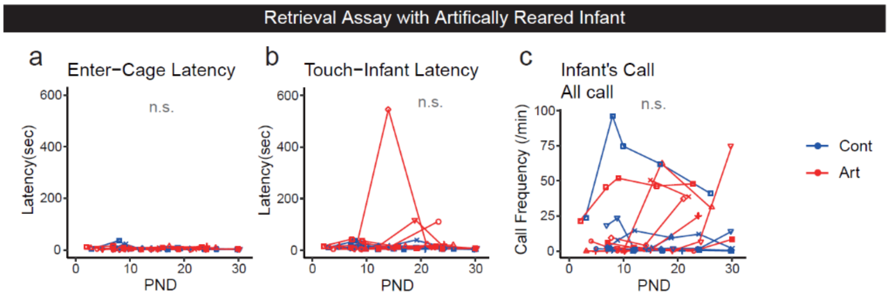

Supplementary Figure 6 Infant retrieval assays with artificially reared infants.

a-c Caregiver-infant behaviors and interactions during the retrieval assay. The shape of the markers represents each dyad.

a Latency of caregiver's reach to the infant cage.

b Latency of caregiver's touch to the infant.

c Frequency of infant calls.

GLMM. Number of sessions: 69. Number of dyads: 8 each.

Supplementary Tables

	Item	Description
Caregiver's behavior	Carrying	Caregiver letting the infant clung to caregiver's body. Including Holding and Transport.
	Holding	Carrying without caregiver's locomotion
	Transport	Carrying with caregiver's locomotion
	Contact	Physical contact of body parts between infant and caregiver
	Breast feeding	Breast feeding (only by mother)
	Licking	Licking infant
	Grooming	Manipulating infant's fur by hand or teeth
	Rejection	Rolling + Pushing + Biting
	Rolling	Rubbing infant on cage wall/floor during carrying
	Pushing	Pushing or scratching infant during carrying
	Biting	Biting infant during carrying
	Attack	Biting or scratching infant without carrying
	Walking	Walking
	Running	Running
	Jumping	Jumping
	Stereotypy	Repeated vertical circling more than three times
	Self-scratching	Scratching own body parts by hand or limb
	Self-grooming	Grooming own body parts
	Playing with object	Manipulating objects by hand or biting objects
	Marking	Rubbing anogenital area on cage or objects
	Feeding	Masticating food pellets
	Yawning	Yawning
	In stimulus cage	Staying in stimulus cage with basket
	Touching basket	Touching basket
	In basket	Staying in basket
Infant's behavior	Avoidant behavior	Infant voluntarily dismount from caregiver without rejection or more than 9.4 sec after the last rejection; OR Infant does not cling to the caregiver even when it has body contact with caregiver
	Searching for contact	Approaching caregiver while not-being-carried
	Moving alone	Moving alone without aiming at contact with caregiver
Call	Twitter	Rapid series of vocal elements, regularly and closely spaced and each rising swiftly in frequency. Territorial call that can also indicate alertness or agitation within the group. Neutral call.
	Tsik	Short call that rises slightly before dropping straight down to a much lower pitch. Made when marmoset is alarmed. Negative call.
	Phee	Long call with constant pitch. Within-group contact call. Neutural call.
	Trill	Call with cyclically fluctuating frequency. Within-group contact call. Neutral~positive call.
	Ek	Low-pitched and short call that is uttered singly or several in close succession. Indicates mild anxiety. Not observed in infants. Negative call.
	Chirp	Short and quiet call that falls from high to low frequency. Made in friendly/affiliative contexts or with a favored food. Not observed in infants. Positive call.
	Chatter	Low-pitched, harsh and staccato call. Aggressive call. Infant's chatter-like calls were classified as repeated tsiks. Negative call.
	Cry	Long and broadband call used to get attention from other group members. Not observed in caregivers in this study. Negative call.
Social context (time period)	*Alone_BeforeRET*	Infant is not carried before the first retrieval
	Alone_AfterRET	Infant is not carried after the first retrieval (except "*During_Rejection* ")
	During_Rejection	Period from the start of rejection to 9.4 seconds after the end of rejection
	Holding	Infant is carried without caregiver's locomotion (except "*During_Rejection* ")
	Transport	Infant is carried with caregiver's locomotion (except "*During_Rejection* ")
	Carrying	*Holding + Transport*

Supplementary Table 1 List of observed behaviors in the retrieval assay.

Infant Name	Birth No. in parents	Mother Name	Test (PND)	Father Name	Test (PND)	Sibling1 Name	Test (PND)	Sibling2 Name	Test (PND)
Hook	1	Fastener	*(3), 7, 10, 15, 18, 21, 24, 28, 31, 35*	Chuck	*(4), 8, 11, 16, 19, 21, 24, 28, 32, 35*				
Wendy	2	Fastener	3, 5, 7, 11, 15, 19, 27, 34	Chuck	4, 8, 27, 29, 34				
John	3	Fastener	7, 15, 19, 28, 33	Chuck	15, 19, 28, 33				
Cubby	4	Fastener	4, 8, 16, 21, 26, 34	Chuck	3, 5, 9, 22, 28, 33				
Bill	1	Hime	*(2), 9, 16, 23, 30*	Oji	*(5), 13, 19, 26, 33*				
Kate	1	Hime	*(5), 14, 20, 27*	Oji	*(2), 8, 15, 22*				
George	2	Hime	5, 13, 19, 26, 33	Oji	3, 9, 16, 23				
Charlotte	2	Hime	3, 9, 16, 23, 31	Oji	5, 13, 19, 26, 33				
Diana	3	Hime	3, 7, 11, 15, 27	Oji	5, 9, 22, 30				
Henry	3	Hime	5, 9, 13, 22	Oji	3, 7, 27				
Eugenie	4	Hime	3, 5, 7, 9, 11, 13, 15, 17, 19, 23, 24, 29, 32, 36	Oji	3, 5, 23, 24, 32, 36				
Pon	1	Kachan	*(1), 13, 18, 25, 32*	Tochan	*(5), 10, 21, 28, 35*	Maru	*(3), (5), 7, 11, 13, 15, 19, 21, 23, 26, 33*		
Yu	1	Kachan	*(5), 10, 21, 28, 35*	Tochan	*(1), 13, 18, 25, 32*	Maru	*(4), (6), 12, 14, 20, 22, 29*		
Gaku	2	Kachan	3, 10, 17, 25, 31	Tochan	6, 13, 19, 34				
Saku	2	Kachan	6, 13, 19, 27, 34	Tochan	3, 10, 17, 25, 31				
Ataro	1	Mama	7, 14, 21, 28	Tochan	3, 10, 17, 24, 31				
Setaro	1	Mama	(3), 10, 17, 24, 31	Tochan	7, 14, 21, 28, 35				
Stone	1	Lame	*8, 14, 21, 29, 35*	Mogol	*(3), 11, 17, 24, 31*	Beads	*10, 15, 23, 30, 36*	Ribbon	*(4), 13, 18, 25, 32*
Pearl	1	Lame	*(3), 11, 17, 24, 31*	Mogol	*8, 14, 21, 29, 35*	Beads	*(4), 13, 18, 25, 32*	Ribbon	*10, 15, 23, 30*
Mimosa	1	Ume	(3), 10, 23, 27, 31	Beads	8, 16, 24, 29, 35				
Kodemari	1	Ume	7, 16, 24, 29, 35	Beads	3, 10, 23, 27				
Ninniku	2			Beads	2, 23				
Gari	2	Ume	2, 23	Beads	12				
Shirayuri	1	Ribbon	7, 14, 24, 31, 36	Kabu	(4), 11, 19, 26, 33				
Suisen	1	Ribbon	4, 11, 19, 26, 33	Kabu	7, 14, 24, 31, 36				

Supplementary Table 2 List of family-reared infants and their retrieval assay sessions.

The numerals indicate the postnatal day of the infant in the session. Parentheses indicate the first week sessions performed by the caregiver without previous experience of infant retrieval assays and were excluded from the statistical analysis. Italics: indicate the sessions without high-quality vocal recordings, which were excluded from the detailed vocal analyses. Underlines indicate the initial 16 sessions that were censored at 300 sec when the first retrieval did not take place, while the others were censored at 600 sec. Red: female, blue: male. See also [37].

Assay	Item	Description
**Infant retrieval assay**	*RET_Latency*	The latency of the first retrieval
(On site, 30-sec bins)	*Carrying Rate*	The rate at which a caregiver carried an infant
	Rejection Rate	The rate of bins with rejection in carrying bins
**Instantaneous scan sampling of the family cage** (Littermates were not distinguished)	*Scan_Carrying Rate*	The rate at which a caregiver carried any infant
**Continuous family observation**	*Family_Carrying Duration*	Total duration of carrying
(Littermates were not distinguished)	*Family_Carrying Bout Duration_Median*	The median duration of a carrying bout
	Family_Rejection Rate	The rate of bins with rejection in carrying bins
**Food transfer assay**	*FS_Transfer Ratio*	The ratio at which an infant was given a food item by a caregiver when the infant approached the caregiver with the food item

Supplementary Table 3 List of parameters employed in our previous paper.
These parameters were obtained in [37] and were used for the correlation matrix in Fig. 1c.

Supplementary Table 4 The r values of the correlation matrix in Fig. 1c.
Red and blue indicate positive and negative correlations, respectively.
(This table is provided as a separate file.)

Supplementary Table 5 The p values of the correlation matrix in Fig. 1c.
Red: $p < 0.05$.
(This table is provided as a separate file.)

Name	Sex	Family (Father) Name	Family Cage	Retrieval Assay	Vocal Recording in Isolation	Reason of Artificial Rearing	Housing condition	Comment
Michael	M	Chuck		✓	✓	Born as a twin, but one nipple of the mother did not work. We have tried to expose and reunite with the original family, but did not succeed, at least partly because both parents were inherently rejective toward any infants. We stopped indirect family exposure through a mesh wall after Michael's finger was bitten by the family member at the mesh.	Singly housed in an incubator in the same breeding room with their family and other marmosets, with occasional exposure to the family. Contacted with a family caregiver during infant retrieval assays (10-15 min, 0-3 times /week).	
GeorgeD	M	Chuck	✓	✓		Born as a twin but one nipple of the mother did not work.	Singly housed in an incubator in the same breeding room with its family. Reintroduced repeatedly to the family (2-6hr/day, 3-7times/week)	
Atako	F	Chuck	✓	✓		Triplet	Singly housed in an incubator in the same breeding room with their family and step family (Chuck). Reintroduction to Chuck's family (2-6hr/day, 3-7times/week)	Chuck's family was assigned for the family exposure for Atako, because the biological family was engaged in the other experiment.
Senazo	M	Yukita	✓			triplet	Singly housed in an incubator in a separate room from its family. Reintroduced repeatedly to the family (3-6hr/day, 3-7times/week)	
Senako	F	Yukita	✓	✓		triplet	Singly housed in an incubator in a separate room from its family. Reintroduced repeatedly to the family (3-6hr/day, 3-7times/week)	

Supplementary Table 6 List of artificially reared infants.

Name	Reared Condition	Sex	DOB	Family (Father) Name	Num. of Elder Siblings	Date of Experiments		PND		Num. of Experiments	Cage Size
						From	To	From	To		
Shirizo	Cont	M	2017/11/17	Yukita	1	2017/11/18	2018/1/9	1	53	15	42 × 65 × 60 cm
Yoshie	Cont	F	2017/11/17	Yukita	1	2017/11/18	2018/1/9	1	53	15	42 × 65 × 60 cm
Senazo	Art	M	2017/11/17	Yukita	1	2017/11/18	2018/1/9	1	53	15	42 × 65 × 60 cm
Nana	Cont	F	2019/6/15	Chuck	3	2019/6/17	2019/7/29	2	44	9	43 × 66 × 60 cm
GeorgeD	Art	M	2019/6/15	Chuck	3	2019/6/17	2019/7/29	2	44	9	43 × 66 × 60 cm
Atako	Art	F	2019/6/16	Chuck	3	2019/6/17	2019/7/12	1	26	6	43 × 66 × 60 cm
Shirie	Cont	F	2019/10/10	Yukita	1	2019/10/11	2019/11/22	1	43	9	42 × 65 × 60 cm
Yoshimi	Cont	F	2019/10/10	Yukita	1	2019/10/11	2019/11/22	1	43	9	42 × 65 × 60 cm
Senako	Art	F	2019/10/10	Yukita	1	2019/10/11	2019/11/22	1	43	9	42 × 65 × 60 cm

Supplementary Table 7 List of family reunion observations with Art infants.

Cont: Control, Art: Artificially reared.

				Father		Mother		Date of Experiments		PND		
Name	Reared Condition	Sex	DOB	Name	Num. of Experiments	Name	Num. of Experiments	From	To	From	To	Cage Size
Cubby	Cont	M	2016/3/31	Chuck	2	Fastener	3	2016/4/14	2016/4/21	14	21	43 × 66 × 60 cm each
Michael	Art	M	2016/3/31	Chuck	2	Fastener	3	2016/4/8	2016/4/22	8	22	43 × 66 × 60 cm each
Nana	Cont	F	2019/6/15	Chuck	5	Fastener	4	2019/6/18	2019/7/11	3	26	43 × 66 × 60 cm each
GeorgeD	Art	M	2019/6/15	Chuck	4	Fastener	4	2019/6/18	2019/7/11	3	26	43 × 66 × 60 cm each
Atako	Art	F	2019/6/16	Chuck	5	Fastener	4	2019/6/18	2019/7/9	2	23	43 × 66 × 60 cm each
Shirie	Cont	F	2019/10/10	Yukita	6	Yuki	5	2019/10/13	2019/11/9	3	30	42 × 65 × 60 cm each
Yoshimi	Cont	F	2019/10/10	Yukita	6	Yuki	5	2019/10/13	2019/11/9	3	30	42 × 65 × 60 cm each
Senako	Art	F	2019/10/10	Yukita	6	Yuki	5	2019/10/13	2019/11/9	3	30	42 × 65 × 60 cm each

Supplementary Table 8 List of retrieval assays with Art infants.

Cont: Control, Art: Artificially reared. “Cage size” represents the size of a home cage. In the test, two home cages were connected by the tunnel.

Supplementary Movies
The movies were provided as separate Supplementary Movie files.

Supplementary Movie 1 Typical retrieval in the retrieval assay
Wendy (PND 15) was left alone and called severely, and then Fastener (mother) retrieved Wendy. After the retrieval, Wendy stopped calling.

Supplementary Movie 2 Caregiver's rejection in the retrieval assay
Gaku (PND 13) was carried and then rejected by Tochan (father). After the rejection, Gaku dismounted and called intensely while alone.

Supplementary Movie 3 Direct transfer of family reunion observation
Yoshie (PND 1) was rejected by Junior (elder brother) and called severely. Yukita (father) approached them (0:18) and then received Yoshie from Junior (0:23). After Yukita's retrieval, Yoshie stopped calling.

Supplementary Movie 4 **Dismounting of infants during carrying in family reunion observation**
The Art infant Senazo (PND 23) clung to the father (Yukita) (0:14) and soon dismounted without distinct rejection (0:23). Then, the Cont littermate Yoshie clung to Yukita (0:32) and was rejected (0:40~). Yoshie kept clinging during the rejection for more than 50 sec and finally dismounted (not included in this video).

Supplementary Movie 5 Avoidant behavior of an artificially reared infant in the retrieval assay
The Art infant Michael (PND 15) clung to the trunk of the father Chuck and dismounted without distinct rejection (0:19). Chuck tried to retrieve Michael again (0:31), but Michael refused to cling. This movie is from the session at the bottom of Fig. 5a (approximately 150-190 sec).

Supplementary Movie 6 First retrieval of a control infant in the retrieval assay
After the first retrieval, the Cont infant (Cubby, the littermate of Michael, PND 15) quickly moved to the back of the father (Chuck).

Supplementary Movie 7 First retrieval of an artificially reared infant in the retrieval assay
After the first retrieval, the Art infant Michael (PND 15) moved onto the body of the father (Chuck) (0:42~). Michael occasionally clung to Chuck's head and face, which induced rejection by Chuck (0:55~). Michael took a longer time to settle to a suitable position than Cubby.
