# Supplementary material for: Anxious about rejection, avoidant of neglect: Infant marmosets tune their attachment based on individual caregiver’s parenting style
Source: Commun Biol. 2024 Feb 20;7:212. doi: 10.1038/s42003-024-05875-6 (PMC10879543; doi:10.1038/s42003-024-05875-6)
Supplement: Supplementary file 3 — Description of Additional Supplementary Files [file 42003_2024_5875_MOESM3_ESM.pdf]

Description of Additional Supplementary Files

File name: Supplementary Data 1
Description: The source data behind the graphs in the paper.

File name: Supplementary Tables 4 and 5
Description: The r & p values of the correlation matrix in Fig 1c. Red and blue indicate positive and negative correlations (Supplementary Table 4). Red: $p < 0.05$ (Supplementary Table 5). Supplementary Table 4 & 5

File name: Supplementary Movie 1
Description: Typical retrieval in the retrieval assay. Wendy (PND 15) was left alone and called severely, and then Fastener (mother) retrieved Wendy. After the retrieval, Wendy stopped calling.

File name: Supplementary Movie 2
Description: Caregiver's rejection in the retrieval assay. Gaku (PND 13) was carried and then rejected by Tochan (father). After the rejection, Gaku dismounted and called intensely while alone.

File name: Supplementary Movie 3
Description: Direct transfer of family reunion observation. Yoshie (PND 1) was rejected by Junior (elder brother) and called severely. Yukita (father) approached them (0:18) and then received Yoshie from Junior (0:23). After Yukita's retrieval, Yoshie stopped calling.

File name: Supplementary Movie 4
Description: Dismounting of infants during carrying in family reunion observation. The Art infant Senazo (PND 23) clung to the father (Yukita) (0:14) and soon dismounted without distinct rejection (0:23). Then, the Cont littermate Yoshie clung to Yukita (0:32) and was rejected (0:40~). Yoshie kept clinging during the rejection for more than 50 sec and finally dismounted (not included in this video).,

File name: Supplementary Movie 5
Description: Avoidant behavior of an artificially reared infant in the retrieval assay. The Art infant Michael (PND 15) clung to the trunk of the father Chuck and dismounted without distinct rejection (0:19). Chuck tried to retrieve Michael again (0:31), but Michael refused to cling. This movie is from the session at the bottom of Fig. 5a (approximately 150-190 sec).

File name: Supplementary Movie 6
Description: First retrieval of a control infant in the retrieval assay. After the first retrieval, the Cont infant (Cubby, the littermate of Michael, PND 15) quickly moved to the back of the father (Chuck).

File name: Supplementary Movie 7

Description: First retrieval of an artificially reared infant in the retrieval assay. After the first retrieval, the Art infant Michael (PND 15) moved onto the body of the father (Chuck) (0:42~). Michael occasionally clung to Chuck's head and face, which induced rejection by Chuck (0:55~). Michael took a longer time to settle to a suitable position than Cubby.
