# Supplementary material for: Anxious about rejection, avoidant of neglect: Infant marmosets tune their attachment based on individual caregiver’s parenting style
Source: Commun Biol. 2024 Feb 20;7:212. doi: 10.1038/s42003-024-05875-6 (PMC10879543; doi:10.1038/s42003-024-05875-6)
Supplement: Supplementary file 13 — Reporting Summary [file 42003_2024_5875_MOESM13_ESM.pdf]

nature portfolio

Corresponding author(s): Atsuko Saito and Kumi O. Kuroda

Last updated by author(s): Jan 9, 2024

Reporting Summary

Nature Portfolio wishes to improve the reproducibility of the work that we publish. This form provides structure for consistency and transparency in reporting. For further information on Nature Portfolio policies, see our Editorial Policies and the Editorial Policy Checklist.

Statistics

For all statistical analyses, confirm that the following items are present in the figure legend, table legend, main text, or Methods section.

n/a	Confirmed	
☐	☒	The exact sample size (n) for each experimental group/condition, given as a discrete number and unit of measurement
☐	☒	A statement on whether measurements were taken from distinct samples or whether the same sample was measured repeatedly
☐	☒	The statistical test(s) used AND whether they are one- or two-sided<br>*Only common tests should be described solely by name; describe more complex techniques in the Methods section.*
☐	☒	A description of all covariates tested
☐	☒	A description of any assumptions or corrections, such as tests of normality and adjustment for multiple comparisons
☐	☒	A full description of the statistical parameters including central tendency (e.g. means) or other basic estimates (e.g. regression coefficient) AND variation (e.g. standard deviation) or associated estimates of uncertainty (e.g. confidence intervals)
☐	☒	For null hypothesis testing, the test statistic (e.g. F, t, r) with confidence intervals, effect sizes, degrees of freedom and P value noted<br>*Give P values as exact values whenever suitable.*
☒	☐	For Bayesian analysis, information on the choice of priors and Markov chain Monte Carlo settings
☒	☐	For hierarchical and complex designs, identification of the appropriate level for tests and full reporting of outcomes
☐	☒	Estimates of effect sizes (e.g. Cohen's d, Pearson's r), indicating how they were calculated

Our web collection on statistics for biologists contains articles on many of the points above.

Software and code

Policy information about availability of computer code

Data collection	For behavioral analysis, video cameras (HDR-AS100V, Sony), a microphone (MKH416-P48U3, Sennheiser) and a linear PCM recorder (DR-60DMKII, TASCAM) were used for video and sound recording.
Data analysis	Vocal and behavioral analysis were conducted using PRAAT (ver. 6.0.43) and Solomon Coder (ver. 19.08.02), respectively. Statistics were performed using software R (R Development Core Team, 2020).

For manuscripts utilizing custom algorithms or software that are central to the research but not yet described in published literature, software must be made available to editors and reviewers. We strongly encourage code deposition in a community repository (e.g. GitHub). See the Nature Portfolio guidelines for submitting code & software for further information.

Data

Policy information about availability of data

All manuscripts must include a data availability statement. This statement should provide the following information, where applicable:

- Accession codes, unique identifiers, or web links for publicly available datasets
- A description of any restrictions on data availability
- For clinical datasets or third party data, please ensure that the statement adheres to our policy

The authors declare that all data supporting the findings of this study are available within the paper.

March 2021

Field-specific reporting

Please select the one below that is the best fit for your research. If you are not sure, read the appropriate sections before making your selection.

☒ Life sciences ☐ Behavioural & social sciences ☐ Ecological, evolutionary & environmental sciences

For a reference copy of the document with all sections, see nature.com/documents/nr-reporting-summary-flat.pdf

Life sciences study design

All studies must disclose on these points even when the disclosure is negative.

Sample size	No statistical methods were used to determine sample sizes. We made an effort to reduce number of subjects as a primate study. Sample size for each experiment was described in the figure legends.
Data exclusions	The data listed in Table S1, S6-8 were included. The criteria for data exclusion were described in the method.
Replication	The data were from at least three independent experiments, showing a similar trend. All attempts at replication were successful. The biological replicate number corresponds to the number of marmosets. Technical replicates were not performed in this study. Cohen's kappa was calculated to evaluate the inter-rater reliability in the behavioral analysis, which confirmed that it was sufficiently consistent. Individual data is presented for transparency.
Randomization	Experiments and observations were conducted with several different marmoset families. To minimize the influence of family and birth, same-birth siblings were used as a control for the artificially reared infants. In some cases, heavier infants were assigned to the artificially reared group to increase survival under artificial rearing, but the difference was small and no significant difference in the later weight was observed.
Blinding	Blinding was not available, because the analyst would identify the individuals. But the inter-rater reliability with another person confirmed the consistency.

Reporting for specific materials, systems and methods

We require information from authors about some types of materials, experimental systems and methods used in many studies. Here, indicate whether each material, system or method listed is relevant to your study. If you are not sure if a list item applies to your research, read the appropriate section before selecting a response.

Materials & experimental systems

n/a	Involved in the study	
☒	☐	Antibodies
☒	☐	Eukaryotic cell lines
☒	☐	Palaeontology and archaeology
☐	☒	Animals and other organisms
☒	☐	Human research participants
☒	☐	Clinical data
☒	☐	Dual use research of concern

Methods

n/a	Involved in the study	
☒	☐	ChIP-seq
☒	☐	Flow cytometry
☒	☐	MRI-based neuroimaging

Animals and other organisms

Policy information about studies involving animals; ARRIVE guidelines recommended for reporting animal research

Laboratory animals	Male and female common marmosets were used.
Wild animals	*Provide details on animals observed in or captured in the field; report species, sex and age where possible. Describe how animals were caught and transported and what happened to captive animals after the study (if killed, explain why and describe method; if released, say where and when) OR state that the study did not involve wild animals.*
Field-collected samples	*For laboratory work with field-collected samples, describe all relevant parameters such as housing, maintenance, temperature, photoperiod and end-of-experiment protocol OR state that the study did not involve samples collected from the field.*
Ethics oversight	All experiments involving marmosets were performed in agreement with guidelines of the Animal Experiment Committee of RIKEN.

Note that full information on the approval of the study protocol must also be provided in the manuscript.
